# Correlation Between Anxiety and Serum Thyroid Hormone Levels in Patients With Papillary Thyroid Carcinoma Undergoing Microwave Ablation

**DOI:** 10.1155/2024/6297567

**Published:** 2024-11-07

**Authors:** Yan Li, Lili Peng, Ying Wei, Zhenlong Zhao, Ming-an Yu

**Affiliations:** Department of Interventional Medicine, China-Japan Friendship Hospital, Beijing, China

**Keywords:** anxiety, microwave ablation, papillary thyroid carcinoma, thyroid-stimulating hormone

## Abstract

**Objective:** This study aims to explore the correlation between anxiety and serum thyroid hormone levels in patients with papillary thyroid carcinoma (PTC) who underwent microwave ablation (MWA).

**Methods:** A total of 107 PTC patients who underwent MWA were recruited. Three months after MWA, serum samples were collected from each participant to analyze their thyroid-related hormone levels, including free T3 (FT3), free T4 (FT4), T3, T4, and thyroid-stimulating hormone (TSH) levels. Additionally, the Self-Rating Anxiety Scale (SAS) was used to measure anxiety levels at the same time. Linear correlation analysis was used to determine the correlation between anxiety and serum thyroid hormone levels.

**Results:** SAS scores among the 107 PTC patients ranged from 27 to 58, with an average score of 38.19 ± 6.68. Seven patients (6.54%) met the criteria for anxiety; the mean SAS score among these patients was 51.57 ± 2.88. The remaining 100 patients (93.46%) did not meet the criteria for anxiety; the mean SAS score among these patients was 37.04 ± 5.49. Among all participants, TSH levels varied from 0.30 to 5.09, with an average of 2.08 ± 0.91. Nine participants exhibited slight fluctuations in FT3, FT4, T3, T4, and TSH levels; these hormone levels consistently remained within the normal range for the other 98 patients. Linear correlation analysis revealed a significant positive correlation between anxiety and TSH levels (*p* < 0.001).

**Conclusion:** The results demonstrated a significant positive correlation between anxiety and TSH levels in patients with PTC post-MWA, indicating that increased anxiety is associated with increased TSH levels.

## 1. Introduction

The global incidence of papillary thyroid carcinoma (PTC) has rapidly risen in recent decades [1, 2]. PTC accounts for more than 95% of all thyroid malignancies and has a good prognosis, with over 90% 10-year overall survival rate [3]. Typically, the standard treatment for PTC patients involves near-total thyroidectomy (TT), followed by postoperative radioiodine therapy [4, 5]. Postoperative thyroid hormone replacement therapy is the mainstay of long-term medical management [6]. Thyroid-stimulating hormone (TSH) acts as a growth factor for thyroid follicular cells, potentially promoting the development and progression of follicular cell–derived thyroid cancers. Maintaining a low serum TSH level could slow the growth and spread of thyroid cancer cells, thereby increasing disease-free and overall survival times [7, 8]. Consequently, postoperative TSH suppression has been proposed as a crucial strategy in thyroid cancer management. However, it is important to note that TSH suppression therapy may lead to side effects, including osteoporosis, atrial fibrillation, sleep disturbances, depression, and anxiety disorders [9–11].

In recent decades, thermal ablation including microwave ablation (MWA) and radiofrequency ablation (RFA) have emerged as alternative methods for managing PTC [12, 13]. These techniques offer several advantages, such as minimal invasiveness and preservation of thyroid function [14, 15], thereby eliminating the need for thyroid hormone replacement therapy in PTC patients following MWA. Additionally, there are no current guidelines recommending TSH suppression after MWA. Given this, exploring alternative methods to manage TSH levels beyond drug therapy becomes necessary. On the one hand, several studies have reported an association between TSH levels and disease progression in PTC patients postoperatively [16–18]. On the other hand, emotional stress is recognized as a significant contributor to PTC [19, 20]. Previous studies have shown that PTC patients frequently exhibit higher levels of anxiety and depression compared to the healthy population, with the prevalence of anxiety ranging from 41.07% to 66.90% [20–23]. This raises the following question: Is there a correlation between anxiety and TSH levels in PTC patients following MWA?

Therefore, investigating the correlation between emotional states and TSH levels could provide valuable reference, potentially leading to the management of TSH levels through emotional interventions rather than drug therapy. In the present study, a linear correlation analysis was conducted to investigate the relationship between anxiety and TSH levels in PTC patients post-MWA.

## 2. Materials and Methods

### 2.1. Participants

This retrospective study was approved by the Institutional Review Board (the number is S2019-283-02). All participants consented to publishing their examination results and provided written informed consent prior to participation in the study. All study procedures were performed in accordance with the principles of the Declaration of Helsinki.

This study consecutively recruited 107 PTC patients between July 2022 and July 2023. Thyroid hormone tests were performed 3 months after MWA, and the Self-Rating Anxiety Scale (SAS) questionnaires were completed at the same time. The inclusion criteria were as follows: (a) pathological diagnosis of PTC; (b) age between 18 and 60 years; (c) able to understand and complete the questionnaire independently; and (d) regular sleep patterns and no history of severe insomnia within the past 3 months. The exclusion criteria were as follows: (a) other concurrent malignancies or other diseases related to hyperthyroidism or hypothyroidism; (b) a history of severe mental illness or cognitive impairment; (c) experiencing psychological trauma or having abnormal interpersonal relationships within the past 3 months; (d) undergoing TSH suppression therapy; (e) taking medications or undergoing medical examinations that could affect thyroid function, such as contrast-enhanced CT scans; and (f) a history of Hashimoto's thyroiditis.

### 2.2. Data Collection

The sociodemographic characteristics included age, sex, education status, marital status, and employment status. Five milliliters of venous blood were collected from each participant using vacuum blood collection tubes 3 months after MWA. The laboratory center of the hospital was responsible for the blood collection and test. The measurement of the serum levels of FT3, FT4, T3, T4, and TSH used a Roche C6000 electrochemiluminescence immunoassay analyzer (Roche Diagnostics, Indianapolis, IN, USA). The normal ranges were 2.0–4.4 pg/mL for FT3, 0.93–1.7 ng/dL for FT4, 0.8–2.0 ng/mL for T3, 5.1–14.1 µg/dL for T4, and 0.27–4.2 µLU/mL for TSH.

### 2.3. Anxiety Evaluation

The SAS includes 20 items that assess a patient's anxiety levels over the past week. Each item is scored on a scale from 1 to 4 points, thus yielding a maximum raw score of 80. The raw score is then multiplied by 1.25 to obtain a standardized total score of 100. Higher scores indicate more severe symptoms of anxiety. The SAS has been shown to have good internal consistency, with a Cronbach alpha coefficient of 0.82 [24].

### 2.4. Statistical Analysis

The Statistical Package for the Social Sciences (SPSS) 26.0 statistical software was used to analyze data and to construct figures. Continuous data are expressed as the mean with standard deviation (M ± SD), and categorical data are presented as counts with percentages (No. [%]). Correlation coefficients were calculated via linear correlation analysis. A *p* value < 0.05 was considered to indicate statistical significance.

## 3. Results

### 3.1. Sociodemographic Characteristics

**Figure 1:**
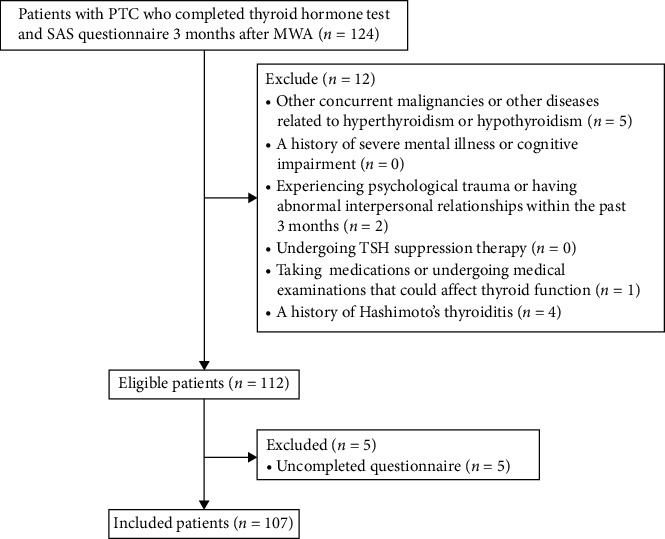
Patient flowchart. MWA, microwave ablation; PTC, papillary thyroid carcinoma; SAS, the Self-Rating Anxiety Scale; TSH, thyroid-stimulating hormone.

A total of 124 PTC patients who underwent MWA completed thyroid hormone tests and the SAS questionnaires 3 months post-MWA. After excluding 17 patients who did not meet the inclusion criteria, 107 patients were included (Figure 1). The sociodemographic characteristics of 107 patients are described in Table 1.

### 3.2. General Outcomes of Anxiety Scores and Serum Thyroid Hormone Levels

Among the 107 PTC patients, the SAS scores ranged from 27 to 58, with a mean score of 38.19 ± 6.68. Seven patients (6.54%) had scores exceeding 49 points (51.57 ± 2.88), which is the cutoff for anxiety [25]. In contrast, 100 patients (93.46%) scored less than 50 points (37.04 ± 5.49), indicating no anxiety. Among all patients, TSH levels ranged from 0.30 to 5.09, with a mean TSH level of 2.08 ± 0.91. In 98 patients (91.59%), the levels of FT3, FT4, T3, T4, and TSH were all consistently within the normal range. Nine patients (8.41%) exhibited slight fluctuations in FT3, FT4, T3, T4, and TSH levels.

The mean levels of FT3 and FT4 in the anxiety group were 1.17 ± 0.12 and 3.22 ± 0.16, respectively, compared to 1.24 ± 0.16 and 3.11 ± 0.37 in the nonanxiety group (*p*=0.16 and *p*=0.15). The mean TSH levels in the anxiety and nonanxiety groups were 3.55 ± 0.97 and 1.98 ± 0.82, respectively (*p*=0.005). Therefore, there were significant differences in TSH levels between the anxiety and nonanxiety groups (Table 2).

### 3.3. Correlations Between TSH, FT4, FT3, T3, and T4 Levels and Anxiety

**Figure 2:**
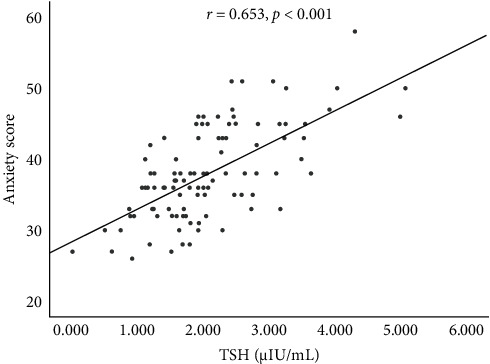
Correlation between TSH levels and anxiety in PTC patients who underwent MWA. MWA, microwave ablation; PTC, papillary thyroid carcinoma; TSH, thyroid-stimulating hormone.

**Figure 3:**
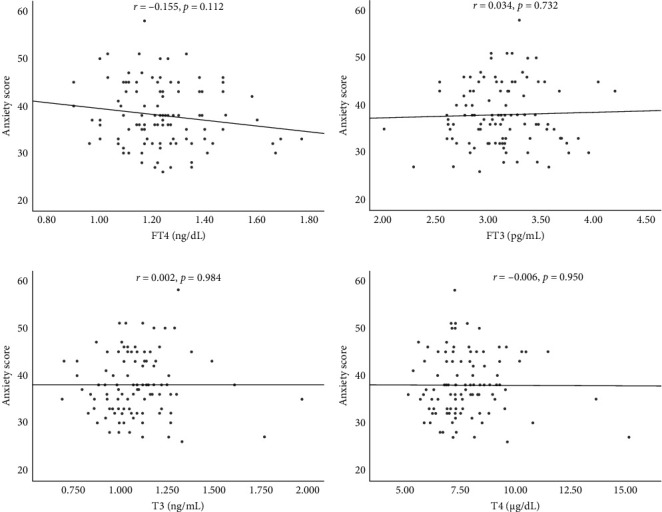
Correlations between (A) FT4, (B) FT3, (C) T3, and (D) T4 levels and anxiety in PTC patients who underwent MWA. MWA, microwave ablation; PTC, papillary thyroid carcinoma.

TSH levels (Figure 2) were positively correlated with SAS scores (*p*  < 0.001), while FT4 (Figure 3A), FT3 (Figure 3B), T3 (Figure 3C) and T4 (Figure 3D) levels were not correlated with SAS scores (*p*=0.112, *p*=0.732, *p*=0.984, and *p*=0.950, respectively). These results indicate a specific positive correlation between TSH levels and anxiety but no significant correlations between FT3 or FT4 levels and anxiety.

## 4. Discussion

Several studies have explored the relationship between emotions and TSH levels; the findings have been inconsistent [26, 27]. Moreover, no clinical studies have yet examined the relationship between TSH levels and anxiety in patients with PTC following MWA.

In the present study, the results revealed that among the 107 PTC patients, SAS scores ranged from 27 to 58, with an average SAS score of 38.19 ± 6.68. Seven patients exhibited anxiety, while the other 100 patients were categorized into the nonanxiety group. TSH levels ranged from 0.30 to 5.09, with an average TSH level of 2.08 ± 0.91. There was a positive correlation between anxiety and TSH levels, suggesting that higher levels of anxiety are associated with higher TSH levels. This finding is consistent with a previous study indicating a positive association between TSH levels and anxiety [28].

Several factors may influence the fluctuations in TSH levels, with anxiety being one significant contributor. According to our analysis, anxiety may influence TSH levels in the following ways. First, anxiety can cause the sympathetic nervous system to experience a relatively heightened state of excitement, leading to increased body metabolism [29, 30]. This heightened metabolism, in turn, increases the thyroid gland burden, potentially inducing increased TSH levels through a negative feedback mechanism. Second, patients with anxiety often experience sleep-related issues, including reduced sleep duration and diminished sleep quality [31]. This poor sleep quality can lead to a decrease in basal metabolism and an increased burden on the thyroid, potentially resulting in elevated TSH levels.

According to the literature, MWA does not influence the thyroid function of PTC patients [15], and there are no recommendations for hormone replacement therapy post-MWA. Moreover, elevated TSH levels may negatively impact disease progression. Therefore, based on the present results, managing and intervening in emotional aspects could be beneficial for maintaining lower TSH levels. This finding offers a potential alternative approach for managing TSH in PTC patients after MWA.

There are a few limitations in the present study. First, the sample size was relatively small, and further studies with larger samples could yield more definitive conclusions. Second, this study exclusively focused on anxiety, neglecting other negative emotions that may also have a potential impact on TSH levels. Therefore, a broader range of emotional indicators should be considered in future research.

## 5. Conclusions

Our findings indicate a positive correlation between anxiety and TSH levels. Emotional management and interventions aimed at alleviating anxiety could play a significant role in maintaining stable TSH levels in PTC patients after MWA.

## Figures and Tables

**Table 1:** Sociodemographic characteristics of PTC patients.

Parameter	*N*	(%)
Sex		
Female	77	71.96
Male	30	28.04
Age		
<45 years	70	65.42
45–60 years	37	34.58
Marital status		
Single	22	20.56
Married	85	79.40
Education status		
Primary/below	1	0.94
Middle/high school	23	21.50
College	56	52.34
College above	27	25.23

Abbreviation: PTC, papillary thyroid carcinoma.

**Table 2:** Comparison of TSH, FT3, and FT4 levels between the anxiety group and nonanxiety group.

Parameter	Anxiety	Nonanxiety	*p* Value
TSH (μLU/mL)	3.55 ± 0.97	1.98 ± 0.82	0.005
FT4 (pg/mL)	3.22 ± 0.16	3.11 ± 0.37	0.15
FT3 (ng/dL)	1.17 ± 0.12	1.24 ± 0.16	0.16

Abbreviation: TSH, thyroid-stimulating hormone.

## Data Availability

The data of this study would be available upon reasonable request and with approval of China-Japan Friendship Hospital. More information on making this request can be obtained from the corresponding author, Ming-an Yu (yma301@163.com).
